# Performance comparison of large language models on pediatric dentistry questions in the Turkish dentistry specialization examination

**DOI:** 10.1186/s12909-025-08315-z

**Published:** 2025-12-29

**Authors:** Hatice Kübra Başkan, Beyhan Başkan

**Affiliations:** 1 Department of Pediatric Dentistry, Faculty of Dentistry, Kahramanmaras Sutcu Imam University, Onikisubat, Kahramanmaras, Türkiye; 2 Department of Endodontics, Faculty of Dentistry, Kahramanmaras Sutcu Imam University, Onikisubat, Kahramanmaras, Türkiye

**Keywords:** Artificial intelligence, Large language models, Pediatric dentistry, Dentistry specialization examination, Performance evaluation

## Abstract

**Background/purpose:**

This study aimed to compare the performance of seven leading large language models (Gemini 2.5 Pro, Grok-4, GPT-5, Claude-4, Copilot, Perplexity, and GPT-4o) on pediatric dentistry questions from the Turkish Dentistry Specialization Examination (DUS), and to identify differences in their performance on information-based versus case-based question types.

**Materials and methods:**

Seven large language models (Gemini 2.5 Pro, Grok-4, GPT-5, Claude-4, Copilot, Perplexity, and GPT-4o) were evaluated on 127 multiple-choice questions from the DUS pediatric dentistry question bank (2012–2021), classified by experts as information-based (*n* = 96) and case-based (*n* = 31). Questions were input in Turkish without modification, and responses were assessed against official answer keys.

**Results:**

Significant differences were observed in overall accuracy rates (*p* < 0.001). The highest overall accuracy was recorded for Gemini 2.5 Pro (94.5%; 120/127), while the lowest performance was seen with GPT-4o (63.0%; 80/127). For information-based questions, Gemini answered 92/96 correctly (95.8%) and GPT-4o 66/96 (68.7%); for case-based questions, Gemini answered 28/31 correctly (90.3%) and Perplexity 5/31 (16.1%). Pairwise Wilcoxon comparisons statistically supported Gemini’s significant superiority over many models and the notably weak performance of GPT-4o and Perplexity on case-based questions (*p* < 0.001).

**Conclusions:**

LLMs can serve as effective “co‑pilots” for information retrieval and exam preparation in dental education but are currently unreliable for diagnostic and treatment decision‑making. Clinicians and students should use LLM outputs for review and learning while retaining final decisions based on professional experience, ethical responsibility, and patient‑centered judgment. Future research should evaluate and enhance LLMs’ multimodal and visual‑data processing capabilities to improve clinical applicability.

## Introduction

In recent years, artificial intelligence, and particularly large language models (LLMs), have shown the potential to revolutionize many professional fields, including medicine and dentistry. These models are used in a wide range of applications, from clinical decision support systems and patient education to professional training and scientific research [1, 2]. Particularly in fields that require a high level of specialized information, such as medicine and dentistry, the performance of LLMs on standardized exams has become an important benchmark for objectively evaluating their knowledge level and clinical reasoning abilities [3].

Pediatric dentistry is a specialized field of dentistry that encompasses multi-layered and interrelated decision-making processes, including growth and development dynamics, anatomical and physiological variations specific to the mixed dentition period, the management of dental trauma, determination of indications for pulp therapy, and behavior guidance strategies for pediatric patients [4]. The multifaceted nature of this discipline necessitates the concurrent application of information-based principles of diagnosis and treatment with clinical reasoning. In this context, the potential use of artificial intelligence-based LLMs in the field of pediatric dentistry offers opportunities. However, for these technologies to function reliably as clinical decision support systems, they must be objectively tested and their performance quantitatively measured using specialist-level question sets and clinical scenarios specific to pediatric dentistry [5, 6] .

The Dentistry Specialization Examination (DUS) is a critical and competitive exam in Turkish that dentists must take to enter specialization programs, measuring in-depth knowledge in both basic and clinical sciences [7]. While the general capabilities of LLMs are known, their performance in a specific clinical field such as pediatric dentistry on a national, high-standard examination like the DUS has not yet been comprehensively investigated. At this point, identifying the differences between the accuracy rates of various LLMs and their performance on different question types, such as information-based and case-based, is crucial for understanding the current capabilities and limitations of these Technologies [8].

The existing literature has examined LLM performance in the context of dentistry primarily through designs that focus on a single model (e.g., previous versions of ChatGPT), involve a limited number of questions, or blend heterogeneous disciplines [6, 9]. In addition to the scarcity of comparative inter-model studies [10, 11], dimensions such as (i) the systematic use of nationally standardized exam questions from a single sub-specialty, (ii) the creation of success profiles differentiated by question type (information-based vs. case-based), (iii) the layered analysis of significance levels using non-parametric multiple comparison statistics, and (iv) the simultaneous evaluation of current, premium-access level LLM versions under identical methodological conditions have remained largely as research gaps. These deficiencies may lead to overlooking the risks of cognitive bias and incorrect reinforcement stemming from the unsupervised use of LLMs during exam preparation and early-career learning processes.

The purpose of this study is to systematically evaluate and compare the performance of seven current and leading artificial intelligence language models (Gemini 2.5 Pro, Grok-4, GPT-5, Claude-4, Copilot, Perplexity, and GPT-4o) on pediatric dentistry questions from past years’ DUS. The study aims to identify the strengths and weaknesses of these models by examining not only their overall accuracy rates but also their separate performance on “information-based” questions, which measure memorized information, and “case-based” questions, which require clinical reasoning and analytical skills.

## Materials and methods

This study did not require ethics committee approval as it was not conducted on human or animal subjects. All data used in the research consisted of outputs from artificial intelligence models accessed via public platforms. No patient data or personally identifiable information was used during the study.

In this study, the performance of LLMs in the DUS pediatric dentistry domain was evaluated. To ensure the statistical power of the study, a power analysis was conducted using G*Power software (version 3.1.9.7, Heinrich-Heine-Universität Düsseldorf, Germany), and it was calculated that a minimum of 97 questions were required, with a significance level (α) of 0.05, statistical power (1-β) of 0.80, and an assumed effect size (d) of 0.5. To enhance the comprehensiveness and validity of the findings, a total of 130 multiple-choice questions from the pediatric dentistry section of the DUS (2012–2021) published by Student Selection and Placement Center (ÖSYM) were included in the study.

### Inclusion and exclusion criteria

In this study, 130 multiple-choice pediatric dentistry questions, each with five answer options, from the DUS (2012–2021) published by ÖSYM were used [12]. Questions from 2022 to 2025, along with three canceled questions, were excluded, bringing the total number of questions analyzed to 127. The questions were classified into two categories by a pediatric dentistry specialist with over 8 years of experience in dental student education: information-based (96 questions) and case-based (31 questions). This categorization was performed to better evaluate the models’ performance on different question types.

### LLMs performance evaluation

In this study, the performance of artificial intelligence language models was evaluated based on their correct and incorrect responses to questions in the DUS pediatric dentistry domain. The LLMs investigated in this study include: Gemini 2.5 Pro, Grok-4, GPT-5, Claude-4, Copilot, Perplexity, and GPT-4o.

At the time of the experiment, new accounts were created for each model, and a premium subscription was purchased for each model. The questions were presented to all models in their original Turkish language, as published by ÖSYM, without any additional instructions or prompting. Each question was input directly into the models’ interfaces exactly as it appeared in the test, and all applications responded to the raw questions immediately without requiring special commands. To prevent the repetition of learning biases and performance improvements in the models, responses were requested and recorded only once. The responses were classified as correct or incorrect by two expert dentists, utilizing ÖSYM’s official answer keys.

The performance of artificial intelligence language models was evaluated based on the following criteria:

Number of Correct Answers: The correct responses provided by the models to case-based, information-based, and total questions were analyzed.

Number of Incorrect Answers: The distribution and percentages of the models’ incorrect responses were evaluated.

### Statistical analysis

Statistical analyses were performed using SPSS for Windows 25.0 (IBM Corp., Armonk, NY, USA). Descriptive statistics were used to summarize correct response rates. Since the Shapiro-Wilk test confirmed a non-normal data distribution (*p* < 0.05), non-parametric tests were employed.

The Friedman test was used to determine if overall performance differences among the seven models were statistically significant. Following a significant result, post-hoc pairwise comparisons were conducted using the Wilcoxon Signed-Rank Test. A Bonferroni correction was applied to account for multiple comparisons (21 pairs), setting the significance threshold at *p* < 0.0024. The z-statistic from the Wilcoxon test is reported, where a positive value indicates that the model in the row performed better than the model in the column. Additionally, Chi-square tests of homogeneity were used to compare the proportions of correct and incorrect answers among models for both case-based and information-based question categories. All tests were two-tailed, and the general significance level was set at *p* < 0.05.

## Results

Of the total 127 DUS questions in the pediatric dentistry domain, Gemini 2.5 Pro achieved the highest accuracy rate (94.5%) by correctly answering 120 questions. While Grok-4 was among the high-performing models along with Gemini 2.5 Pro, GPT-4o showed the lowest performance with 80 correct answers and an accuracy rate of 63%. The distribution of correct and incorrect answers is presented in Table 1.

**Table 1:** Overall counts and percentages of correct and incorrect answers by seven artificial intelligence models to DUS questions, with Friedman test statistics and post-hoc comparisons

LLMs	n	Total Response Count		χ²(6)	*p*	Post-hoc
		n	%			
Claude-4	Correct	106^b^	83.5	85.505	< 0.001*	a > b, c ab, b > c
	Incorrect	21	16.5			
Copilot	Correct	101^b^	79.5			
	Incorrect	26	20.5			
Gemini 2.5 Pro	Correct	120^a^	94.5			
	Incorrect	7	5.5			
Grok-4	Correct	114^ab^	89.8			
	Incorrect	13	10.2			
GPT-4o	Correct	80^c^	63			
	Incorrect	47	37			
GPT-5	Correct	107^b^	84.3			
	Incorrect	20	15.7			
Perplexity	Correct	86^c^	67.7			
	Incorrect	41	32.3			

Post-hoc (a, b, c): Shows the results of post-hoc pairwise comparisons (Wilcoxon Signed-Rank Test with Bonferroni correction) performed after a significant Friedman test. Models sharing the same letter are not statistically significantly different from each other (adjusted *p* < 0.0024). There is a significant difference between models with different letters

n: Number of correct and incorrect answers

%: Percentage of correct and incorrect answers for each model

χ²(6): Friedman test statistic for the difference in overall correct/incorrect answer rates across the models

*p < 0.05: Indicates statistical significance at the 0.05 level

The Friedman test revealed a statistically significant difference in the overall performance (correct/incorrect responses) across the seven LLMs, χ²(6) = 85.505, p < 0.001 (this statistic is also presented as χ² under ‘Total Response Count’ for LLMs in Table 1. The questions were categorized for assessment as either information-based (*n* = 96) or case-based (*n* = 31), and the corresponding accuracy rates for each model are summarized in Table 2. In the information-based questions, Gemini 2.5 Pro demonstrated the highest performance by correctly answering 92 out of 96 questions, while GPT-4o achieved the lowest success with 66 correct answers. In the case-based questions, Gemini 2.5 Pro again exhibited the highest performance by correctly answering 28 out of 31 questions, while Perplexity had the lowest accuracy rate (16.1%) with only 5 correct answers, showing the weakest performance in this category. Although GPT-4o had a moderate rate of correct answers on case-based questions, it displayed the lowest performance overall in both information-based questions and all questions combined. Furthermore, for the GPT-4o, Copilot, and Perplexity models, the number of incorrect answers in case-based questions was found to be greater than the number of correct answers.

**Table 2:** Counts and percentages of correct and incorrect answers by seven artificial intelligence models to DUS questions, categorized as case-based and information-based

		Case-based questions		χ²(6)	*p*-value	Information-based questions		χ²(6)	*p*-value
		*n*	%			*n*	%		
Claude-4	Correct	20^ab^	64.5	45.328	< 0.001*	86^ab^	89.6	66.721	< 0.001*
	Incorrect	11	35.5			10	10.4		
Copilot	Correct	11^bc^	35.5			90^a^	93.7		
	Incorrect	20	64.5			6	6.3		
Gemini 2.5 Pro	Correct	28^a^	90.3			92^a^	95.8		
	Incorrect	3	9.7			4	4.2		
Grok-4	Correct	22^ab^	71			92^a^	95.8		
	Incorrect	9	29			4	4.2		
GPT-4o	Correct	14^bc^	45.1			66^c^	68.7		
	Incorrect	17	54.9			30	31.3		
GPT-5	Correct	19^ab^	61.3			88^ab^	91.6		
	Incorrect	12	38.7			8	8.4		
Perplexity	Correct	5^c^	16.1			81^b^	84.4		
	Incorrect	26	83.9			15	15.6		
Post-hoc		a > bc, c; ab > c				a > b, c; ab > c			

Post-hoc (a, b, c): Shows the results of post-hoc multiple comparison analyses performed to determine which models significantly differ from each other when the Chi-square test is significant. Models sharing the same letter are not statistically significantly different from each other (after Bonferroni correction for pairwise comparisons). There is a significant difference between models with different letters (*p* < 0.0024)

n: Number of correct and incorrect answers

%: Percentage of correct and incorrect answers for each model

χ²(6) (Case/Information-based): Chi-square homogeneity test statistic for the difference in correct/incorrect answer rates among the models within the respective question category

*p < 0.05: Indicates statistical significance at the 0.05 level

**Fig. 1:**
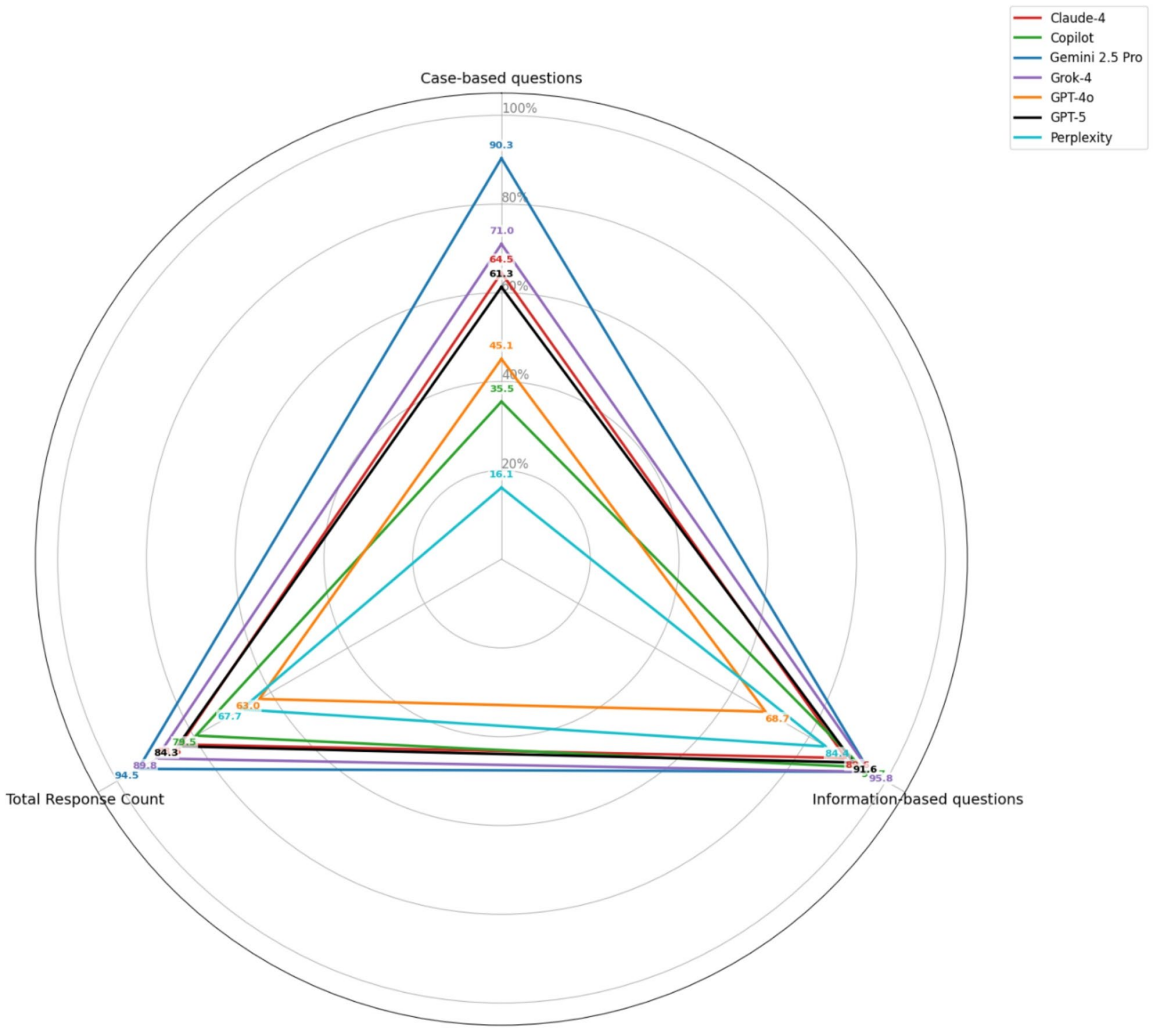
Visualizes the profiles of the examined artificial intelligence models across three key performance metrics: Case-Based Accuracy, Information-Based Accuracy, and Overall Accuracy. Each axis represents a metric, while the area of the polygon enclosed by a model’s plot serves as an indicator of its overall performance

These performance differences are visualized in detail using a radar chart. The radar chart reveals the profile of each artificial intelligence model across three key performance metrics: case-based accuracy, information-based accuracy, and overall accuracy. Axes extending from the center represent the metrics, and the values for each model are connected to form a polygon. A larger area generally indicates better performance. Gemini 2.5 Pro, with the largest polygon area, achieved the highest values on all axes (case-based: 90.3%, information-based: 95.8%, overall: 94.5%), demonstrating its balanced and superior performance. While GPT-4o and Perplexity show low performance in case-based accuracy (45.1% and 16.1%, respectively), they perform better in information-based accuracy (68.7% and 84.4%), revealing a weakness in case-based questions. Following Gemini 2.5 Pro, Grok-4 and GPT-5 exhibit strong and relatively balanced performance (information-based: 95.8% and 91.6%; case-based: 71% and 61.3%, respectively). In contrast, Claude-4 and Copilot demonstrate lower overall accuracy and a particular weakness in case-based questions (case-based: 64.5% and 35.5%). Overall, the radar chart visually illustrates that Gemini 2.5 Pro is the most versatile model, that GPT-4o and Perplexity struggle with case-based questions, and that the other models fall between these two extremes (Fig. 1).

The relationship between LLMs and answer accuracy was substantiated by statistical analyses. Comparisons among the models revealed a statistically significant relationship (*p* < 0.001), and performance differences were evaluated using the Wilcoxon Signed-Rank Test. According to the key findings, significant positive differences in correct answer rates were found between Gemini 2.5 Pro and other models: in comparison to Copilot (z = 3.657, *p* < 0.001), GPT-4o (z = 6.172, *p* < 0.001), and Perplexity (z = 5.667, *p* < 0.001). Furthermore, the Claude-4, Grok-4, and GPT-5 models demonstrated significantly higher correct answer rates compared to the GPT-4o model (Claude-4: z = 4.352, *p* < 0.001; Grok-4: z=-4.529, *p* < 0.001; GPT-5: z = 4.439, *p* < 0.001). Similarly, the Gemini 2.5 Pro, Grok-4, and GPT-5 models exhibited significantly higher correct answer rates in comparison to the Perplexity model (Gemini 2.5 Pro: z = 3.657, *p* < 0.001; Grok-4: z = 3.888, *p* < 0.001; GPT-5: z = 3.656, *p* < 0.001, respectively). All reported pairwise comparisons remained statistically significant after the application of the Bonferroni correction for multiple comparisons (adjusted α = 0.0024). A positive z-value indicates that the model listed in the row performed better than the model in the column. These statistical findings are presented in detail in Table 3.

**Table 3:** Pairwise comparison of LLMs using the Wilcoxon signed-rank test

		Claude-4	Copilot	Gemini 2.5 Pro	Grok-4	GPT-4o	GPT-5	Perplexity
Claude-4	z	1						
	p	-						
Copilot	z	-0.962	1					
	p	0.336	-					
Gemini 2.5 Pro	z	3.441	3.657	1				
	p	0.001*	0.001*	-				
Grok-4	z	0.853	1.333	-2.840	1			
	p	0.394	0.182	0.005	-			
GPT-4o	z	-4.352	-3.015	-6.172	-4.529	1		
	p	0.001*	0.003	0.001*	0.001*	-		
GPT-5	z	0.447	1.177	-3.357	-0.408	4.439	1	
	p	0.655	0.239	0.001*	0.683	0.001*	-	
Perplexity	z	-3.413	-2.402	-5.667	-3.888	1.061	-3.656	1
	p	0.001*	0.016	0.001*	0.001*	0.289	0.001*	-

A positive z-value indicates that the model in the row performed better than the model in the column. Statistically significant differences (indicated by *) are observed when *p* < 0.0024 (Bonferroni corrected for 21 comparisons)

**Fig. 2:**
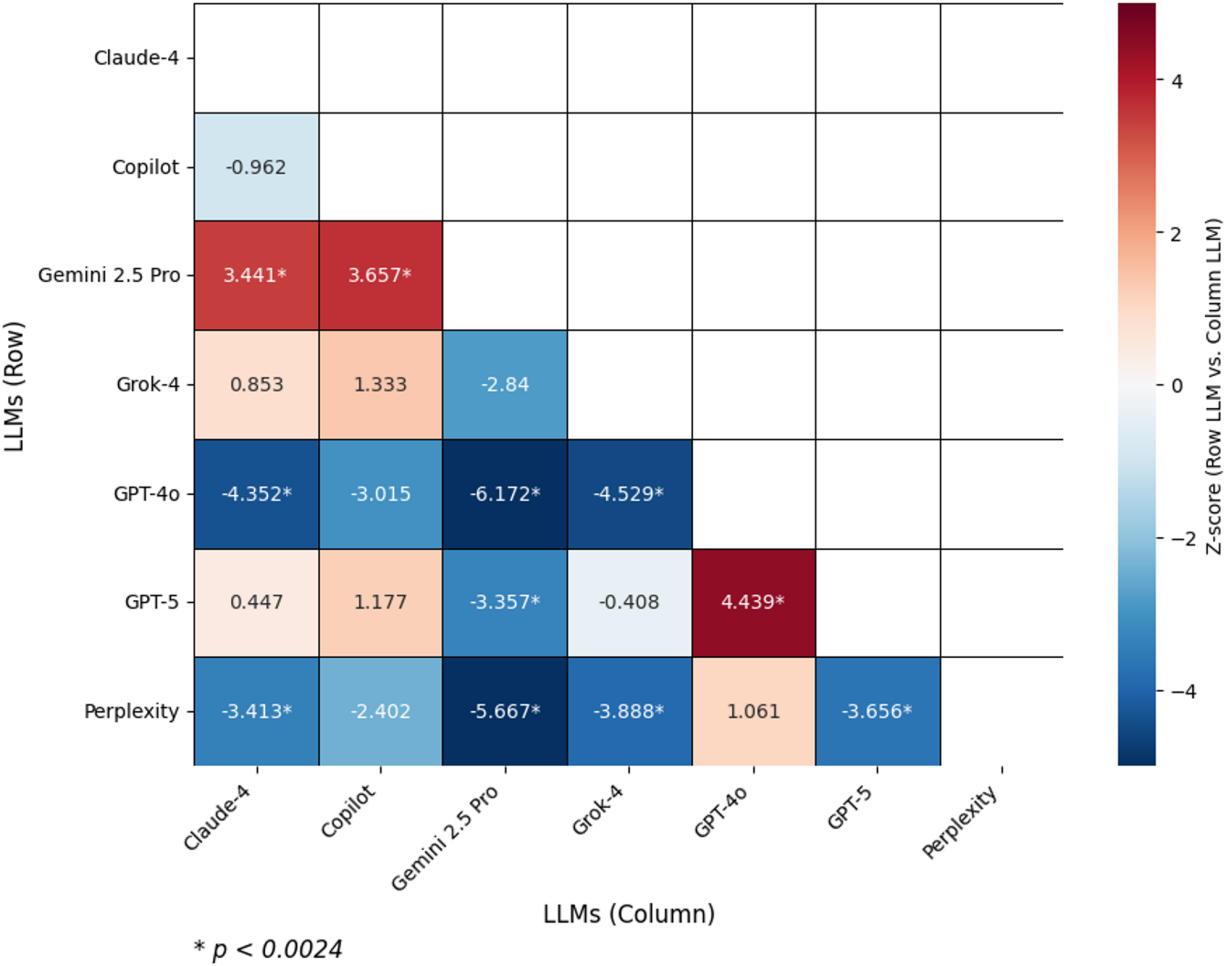
Pairwise performance comparison of Large Language Models (LLMs). The heatmap displays the Z-score for each head-to-head comparison between the LLM in the corresponding row and the LLM in the column. Positive Z-scores (red) indicate that the row LLM outperformed the column LLM, whereas negative Z-scores (blue) indicate the opposite. The intensity of the color corresponds to the magnitude of the performance difference. An asterisk (*) denotes a statistically significant difference at the *p* < 0.0024 level

These statistical differences are visualized in detail through a heatmap (Fig. 2), which illustrates the z-statistics from pairwise Wilcoxon Signed-Rank tests. The color intensity represents the magnitude of the z-statistic, with red tones indicating superior performance of the row model compared to the column model, and blue tones indicating inferior performance. Given the application of the Bonferroni correction (adjusted α = 0.0024), only comparisons exceeding this threshold are considered statistically significant. Gemini 2.5 Pro exhibits superior performance against all other models (e.g., vs. Claude-4: z = 3.441; vs. Copilot: z = 3.657; *p* < 0.001), whereas GPT-4o and Perplexity performed significantly worse than most models (e.g., GPT-4o vs. Grok-4: z=-4.529, *p* < 0.001). Among the other models, Grok-4 and GPT-5 occupy an intermediate position, lagging behind the top performers but outperforming the weaker ones; notably, there is no significant difference between Claude-4 and Copilot (z=-0.962, *p* = 0.336). Overall, the heatmap indicates that Gemini 2.5 Pro is the strongest model, GPT-4o and Perplexity are the weakest, and the remaining models are positioned between these two extremes.

## Discussion

The present study conducted a comparative analysis of the performance of seven contemporary LLMs on pediatric dentistry questions from the Turkish’s DUS. The primary finding of our study is the existence of a statistically significant and distinct performance hierarchy among the LLMs. Gemini 2.5 Pro outperformed all other models with its superior performance in both overall accuracy (94.5%) and in case-based questions requiring clinical reasoning (90.3%). This result is significant as it demonstrates that artificial intelligence technologies have moved beyond mere information storage and retrieval capabilities, reaching the potential to analyze complex clinical scenarios. This study addresses a notable gap in the literature. Whereas previous work has typically concentrated on single-model evaluations or multi-disciplinary question sets [13–16], our research provides a systematic comparison of leading, state-of-the-art models within a single specialized domain, benchmarked against a national standardized exam. Our methodology, employing Turkish-language prompts in a synchronized multi-LLM comparison, aligns with recent approaches in other dental specialties and medical fields, reinforcing the validity of this design [10, 17].

One of our most striking findings is the differentiation in the models’ performance profiles between “information-based” and “case-based” questions. While models such as Gemini 2.5 Pro and Grok-4 exhibit a balanced and high performance across both question types, this balance is disrupted in models like Perplexity and GPT-4o. Particularly, the fact that Perplexity achieves a high success rate of 84.4% on information-based questions while exhibiting an accuracy of only 16.1% on case-based questions suggests that this model’s architecture may be optimized for direct information retrieval and summarization, rather than for the capability of multi-step clinical reasoning and synthesis. This clearly demonstrates that for an LLM, possessing a vast information base does not necessarily mean it can correctly apply that information to solve a clinical problem. The failure in case-based questions indicates that the model is unable to integrate diagnostic criteria, treatment indications, and patient management principles within the context of a scenario. This finding confirms the concerns that the unsupervised use of LLMs, especially in the management of complex cases, could lead to cognitive biases and incorrect reinforcement [18]. Additionally, as also highlighted by Kung et al. [19], the extensive database-driven training process of LLMs can offer a significant advantage when addressing questions based on theoretical information. This indicates that the models’ capabilities for information access and analysis can be used effectively in academic and theoretical contexts. These observations, particularly regarding varied performance across question types and linguistic contexts, resonate with findings from other multi-LLM evaluations on national exams, such as the DUS head and neck anatomy study by Yılmaz et al. [17], further solidifying the need for domain-specific assessment.”

Furthermore, it is important to note that models such as GPT-5 and Grok-4 represent the latest iterations of their respective platforms, with limited prior evaluation in the academic literature, particularly within specialized domains like pediatric dentistry. While our results demonstrate their strong performance, ranking them among the top-tier models in this assessment, this should be interpreted considering their novelty. The absence of extensive prior studies makes direct comparisons challenging, but simultaneously highlights the value of our study in providing some of the first benchmarks for these models in a non-English, specialized context. This may positively influence the perception of their capabilities, as they demonstrate competitive accuracy despite being newer entrants with less domain-specific optimization compared to more established models. However, as with all AI technologies, continuous evaluation will be necessary as these models evolve and more comparative data becomes available.

Other studies in the literature consistently show that ChatGPT-4 and its variants outperform other artificial intelligence models in various medical specialties [10, 11, 20–22]. On the other hand, the fact that GPT-4o demonstrated significantly lower performance (*p* < 0.001) in our study compared to mid- and high-tier models like Claude-4, Grok-4, and GPT-5—in contrast to these findings in the literature—suggests that the generation number in a model’s name does not always guarantee superior performance, and that domain-specific success is closely related to the model’s training data and architecture. Similarly, the mid-tier performance of Bing’s counterpart (Copilot) in our study, in contrast to its top-tier ranking in the Peruvian National Medical Licensing Exam [15], underscores that a model’s superiority is not universal but highly dependent on the specific task, language, and domain.

The domain-dependent performance patterns observed in our study are corroborated by other research within the Turkish DUS context. For instance, a study evaluating eight LLMs on DUS oral pathology questions found that model proficiency significantly varied between case-based and information-based questions, with different models excelling in each category [10] Similarly, another DUS-focused study in prosthodontic dentistry reported that while Gemini provided more accurate answers, the difference was not always statistically significant across all topics [23]. These findings reveal that the performance of LLMs can vary depending on the domain and content of the question being addressed. Collectively, this growing body of DUS-specific evidence, including our own findings in pediatric dentistry, strongly suggests that there is no single universally superior LLM for dental specialization education in Turkey. Instead, performance is highly contextual, depending on the dental sub-discipline, question type, and potentially the linguistic nuances of the original exam questions.

In a previous study that evaluated the performance of three AI chatbots in answering patient FAQs (Frequently Asked Questions) about dental prostheses, Gemini had the highest quality score and was found to significantly outperform Microsoft Copilot. The readability analysis revealed that ChatGPT produced significantly more complex responses compared to Gemini and Copilot. It showed that ChatGPT’s responses were categorized as ‘fairly difficult,’ whereas Gemini’s were ‘plain,’ indicating a significant difference between them. The study concluded that AI chatbots demonstrate significant potential in answering patient questions about dental prostheses, and that improvements are needed to enhance their effectiveness as patient education tools [24]. In another study, the performance of AI chatbots, including GPT-3.5, GPT-4, Bard, Claude, and Bing, was evaluated on the Peruvian National Medical Licensing Exam (P-NLME), and it was reported that the two top-performing models were GPT-4 and Bing [15]. This contrast with our results, where Bing’s counterpart (Copilot) performed in the mid-tier, underscores a critical point: a model’s superiority is not universal but is highly dependent on the specific task, language, and domain. Therefore, our results for DUS pediatric dentistry, combined with these studies, reinforce that benchmarking AI models requires a context-specific framework rather than assuming generalizability from one scenario to another.

A study aimed to evaluate national dental board-style exam questions generated by an LLM (ChatGPT 4o) against those created by human experts using item analysis. The authors reported that the LLM can generate national board-style exam questions of a quality equivalent to those developed by human experts [25]. An earlier study also evaluated neurophysiology exam questions created by ChatGPT and human experts. The findings indicated that the questions generated by ChatGPT were of a similar quality to those written by the experts [26]. Furthermore, it has been reported in the literature that a comparison between ChatGPT and Google Bard (now Gemini) for the purpose of creating multiple-choice questions on dental caries showed that both models exhibited similar performance; however, the questions generated by Gemini possessed higher cognitive levels [27]. Collectively, these findings, alongside our own, paint a picture of LLMs as dual-purpose tools in dental education: reliable for assessing knowledge (as shown in our study) and capable of generating it. This convergence of capabilities underscores their potential for creating comprehensive, AI-augmented educational ecosystems, where models can both generate practice questions and reliably evaluate student responses, particularly in specialized domains like pediatric dentistry.

The potential contribution of LLMs to students’ diagnostic learning processes also requires consideration. A study by Gokkurt Yılmaz et al. [28] showed that personalized feedback generated by ChatGPT‑4o based on Medical Subject Headings (MeSH) significantly improved dental students’ radiographic diagnostic performance compared with traditional correct/incorrect feedback methods. This result indicates that LLMs can be used not only as assessment tools but also as effective instructional aides that identify and target learning gaps. Therefore, these technologies in pediatric dentistry education may actively support the development of diagnostic skills by offering student‑specific guidance rather than merely testing knowledge.

The use of artificial intelligence in healthcare offers the ability to collect and integrate information from various data sources, such as electronic dental and medical records. Through programmed algorithms, it provides significant contributions in areas such as offering evidence-based guidelines to clinicians, supporting diagnosis and treatment planning, assisting in surgical interventions, and performing surgical procedures. Additionally, it supports educational processes by providing students with clinical simulations in a safe and controlled environment [29, 30]. Given the potential of artificial intelligence to enhance efficiency in healthcare, Kim et al. emphasize that dental educators have a responsibility to prepare their students to become professionals who use AI technology responsibly and effectively in their clinical decision-making processes [31]. This responsibility now extends to guiding students on how to critically evaluate and interact with LLMs, based on an informed understanding of their specific strengths and limitations, as quantified by studies like ours.

Based on the data from the current study and other data in the literature, dental educators and students should understand the potential and current risks of LLMs; they should use these technologies as a “co-pilot,” but must not forget that the final clinical decision must always be based on professional information, experience, and ethical responsibility [32–34].

Our study also has some limitations. Firstly, the assessment was conducted using only text-based multiple-choice questions; visual data, such as radiographs, clinical photographs, or laboratory findings, which are frequently encountered in actual clinical practice, were outside the scope of the analysis. Secondly, the performance of the models may be sensitive to the “prompt” strategy used; different prompt engineering techniques could potentially improve the performance of some models. Thirdly, this study reflects a “snapshot” of a specific moment in the development of LLMs; since these models are constantly updated, the performance rankings may change in the future. Finally, the study was conducted in the Turkish language and in the context of an exam specific to Türkiye; the generalizability of the results to other languages or different educational systems requires further research.

## Conclusion

This study compared the performance of seven leading LLMs (Gemini 2.5 Pro, Grok‑4, GPT‑5, Claude‑4, Copilot, Perplexity, and GPT‑4o) on DUS pediatric dentistry questions and found Gemini 2.5 Pro to be the best with an overall accuracy rate of 94.5%, while Grok‑4 and GPT‑5 showed balanced performance. The models were strong on questions requiring theoretical knowledge but showed marked weaknesses on case‑based questions requiring clinical reasoning, highlighting limitations in converting theoretical knowledge into clinical decisions. LLMs can be a useful “co‑pilot” for accessing information and preparing for exams in dental education; however, they are unreliable for diagnostic and treatment decisions. Clinicians and students should use LLMs for information retrieval and review while making final decisions based on professional experience, ethical responsibility, and patient‑centered judgment. Future studies should aim to make them more suitable for clinical practice by testing AI systems’ capabilities for processing visual data and their multimodal abilities.

## Data Availability

The datasets used and/or analysed during the current study are available from the corresponding author on reasonable request.
